# Effect of the topical application of an ethanol extract of quince seeds on the development of atopic dermatitis-like symptoms in NC/Nga mice

**DOI:** 10.1186/s12906-017-1606-6

**Published:** 2017-01-31

**Authors:** Takeshi Kawahara, Kanako Tsutsui, Eri Nakanishi, Toshifumi Inoue, Yasunori Hamauzu

**Affiliations:** 1 Department of Sciences of Functional Foods, Graduate School of Agriculture, Shinshu University, 8304 Minamiminowa, Kamiina, Nagano, 399-4598 Japan; 2 Research Center for Fungal & Microbial Dynamism, Shinshu University, 8304 Minamiminowa, Kamiina, Nagano, 399-4598 Japan; 3 Jupon International, 4-31-5 Nishikojiya, Ota-ku, Tokyo, 144-0034 Japan

**Keywords:** Quince seed, *Cydonia oblonga*, Atopic dermatitis, Keratinocyte, Thymus- and activation-regulated Chemokine

## Abstract

**Background:**

Quince (*Cydonia oblonga* Miller) is a deciduous shrub belonging to the *Rosaceae* family. Quince seed extract has long been used as a cosmetic ingredient for its moisturizing effect. However, little is known about whether quince seed extract has therapeutic effects on keratinocyte-associated skin inflammation.

**Methods:**

In the present study, we investigated the effect of the topical application of ethanol extract of quince seeds (QSEtE) on atopic dermatitis (AD) symptoms in NC/Nga mice. The direct effect of QSEtE on keratinocytes was evaluated using the human keratinocyte cell line HaCaT.

**Results:**

The preliminary application of QSEtE markedly reduced house dust mite allergen-induced skin lesions. The expression of thymus- and activation-regulated chemokine (TARC) in dorsal skin was downregulated. QSEtE directly suppressed the expression and production of TARC in HaCaT cells.

**Conclusions:**

The results suggest that the topical application of QSEtE is effective in preventing the onset of and ameliorating the atopic symptoms of keratinocyte-associated skin inflammation by suppressing TARC production in keratinocytes.

## Background

Quince (*Cydonia oblonga* Miller) is a deciduous shrub that belongs to the *Rosaceae* family and has been mainly cultivated in Asia and areas of the Mediterranean since ancient times [1]. Although quince fruit is not edible owing to its hard, tough and fibrous consistency, it has been used in honeydew and liquors for its pleasant aroma [2]. In addition, peculiar mucilage, which is a mixture of cellulose and water-soluble polysaccharides, is obtained from quince seeds by soaking them in water [3]. The mucilage is reported to have proliferation-enhancing effect on skin fibroblasts [4], healing effects on incised wounds [5], and protective effects against dermal toxicity caused by T2-toxin [6]. Therefore, the mucilage from quince seeds has long been used as a cosmetic ingredient, known as “quince seed extract”, to maintain the barrier function of the skin [7]. However, little is known about the physiological effect of quince seed extract on skin keratinocytes, and the associated onset of atopic side effects on the skin.

Atopic dermatitis (AD) is a chronic inflammatory skin disease associated with intense pruritus and a series of exacerbations and remissions [8]. AD is recognized as a type I and type IV complex according to the Coombs and Gell classification system [9]. Both T-helper (Th2) type immunologic reactions and T-cell-mediated delayed hypersensitivity are involved in AD. In addition, it has been recognized that the expression of various inflammatory chemokines produced by keratinocytes plays important roles in the pathogenesis of AD [10].

In the present study, we aimed to evaluate whether quince seeds have a suppressive effect on the incidence and development of allergic inflammation in the skin. For this purpose, an AD model mouse and a human keratinocyte cell line were used.

## Methods

### Preparation of QSEtE

The fruits of quince ‘Smyrna’ were purchased from the fruit farm in the Minamiminowa-mura area of Nagano, Japan and were formally surveyed and identified at the Laboratory for Postharvest Science and Functional Properties of Fruits and Vegetables. Twenty grams of seeds was separated from the fruits and immersed in 80 mL ethanol. Then, extraction was performed for 1 week at room temperature. The ethanol extract was then filtered using a Buchner funnel with filter paper discs to remove insoluble residues. Then, the extract was dissolved in water after removal of ethanol and lyophilized using an FD-5 N freeze dryer (EYELA) to obtain powdered QSEtE. We re-constituted QSEtE in ethanol at the indicated concentrations and sterilized using a 0.2-μm pore size cellulose acetate membrane filter (Advantec, Tokyo, Japan) before use.

### Mice

Specific pathogen-free, female NC/Nga mice, aged 10 weeks, with 19–23 g body weight were purchased from Charles Liver Laboratories Japan, Inc. (Kanagawa, Japan). The animals were housed at 23 ± 3 °C under a 12-h light/dark cycle and acclimatized for 7 days in laboratory condition before experiments. Food and water were provided ad libitum. All animal protocols were approved by the Committee for Animal Experiments of Shinshu University (Matsumoto, Japan).

### Induction of dermatitis

**Fig. 1:**
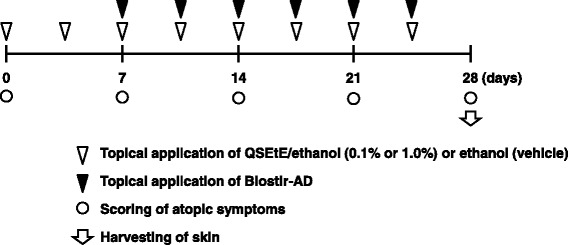
Experimental design of QSEtE application. To induce AD-like symptoms, Biostir-AD was topically applied to the skin of NC/Nga mice twice weekly for 3 weeks. Mice were either treated with QSEtE dissolved in ethanol (0.1 or 1.0% QSEtE), or ethanol alone (vehicle) twice weekly 1 week prior to the application of Biostir-AD application

A schematic procedure of the topical application of QSEtE to NC/Nga mice is shown in Fig. 1. At the start of the experiment, mice were randomly divided into the following three groups (*n* = 6 for each group): ethanol-treated group (vehicle), 0.1% (w/v) QSEtE/ethanol-treated group (0.1% QSEtE), and 1.0% (w/v) QSEtE/ethanol-treated group (1.0% QSEtE). The experimental unit is a cage with a single animal. AD-like skin lesions were induced by topical application of Biostir-AD (Biostir, Kobe, Japan), a hydrophilic petrolatum-based ointment-containing extract of house dust mite (*Dermatophagoides farinae*), according to the manufacturer’s instructions. The hair on the upper dorsal skin and the back of the ears of mice was shaved under isoflurane anaesthesia, and 100 μL of 4% (w/v) sodium dodecyl sulphate was applied to shaved skin for barrier disruption. After 3 h, 100 μL ethanol (vehicle), 0.1% QSEtE in ethanol, or 1.0% QSEtE in ethanol was applied to the area twice a week. Then, the following week, Biostir-AD was applied to the skin area immediately after QSEtE application. The application of the test substrate and Biostir-AD was repeated twice weekly for a further 3 weeks. On day 28 of the experiment, mice were sacrificed by cervical dislocation. Then, the ear and dorsal skin were harvested for histological analyses and analysis of gene expression, respectively. Any clinical signs related to toxicity, such as remarkable loss of body weight, were monitored in all groups of animals throughout the course of experiment.

### Evaluation of the severity of skin lesions

The severity of dermatitis was evaluated according to a previously described method [11]. Briefly, the development of erythema haemorrhage, scarring dryness, oedema and excoriation erosion was scored as 0 (none), 1 (mild), 2 (moderate) and 3 (severe). The sum of the individual scores was taken as the dermatitis score.

### Histopathological studies

The harvested ear of mice was immediately fixed in 10% (v/v) neutral buffered formalin and left for 48 h. Then, the fixed ear was sliced and embedded in paraffin, sectioned, deparaffinised and rehydrated at the Biopathology Institute Co., Ltd. (Oita, Japan). The processed sections were either subjected to hematoxylin and eosin staining to evaluate oedema, or subjected to toluidine blue staining to evaluate infiltrated mast cells.

### Cells

A human keratinocyte cell line, HaCaT, was purchased from Cell Lines Service GmbH (Eppelheim, Germany). HaCaT cells were cultured using high glucose Dulbecco’s modified Eagle’s medium (Wako Pure Chemical Industries, Ltd., Osaka, Japan) supplemented with 10% heat-inactivated foetal bovine serum (Equitech-Bio, Kerrville, TX, USA), 2 M L-glutamine, 100 U/mL penicillin G and 100 μg/mL streptomycin. Cells were passaged using conventional procedures with 0.05% trypsin in a humidified atmosphere of 5% CO_2_/95% air at 37 °C.

### Induction of thymus and activation-regulated chemokine (TARC)

TARC was induced from HaCaT cells according to the method of Sumiyoshi et al. [12]. Briefly, HaCaT cells (2.5 × 10^5^ cells/1 mL/well) were seeded in a 24-well tissue culture plate (Falcon; Corning Incorporated, Corning, NY, USA) and cultured with the aforementioned concentrations of QSEtE for 24 h. The cells were then stimulated with 10 ng/mL human recombinant tumour necrosis factor (TNF)-α (PeproTech, Rocky Hill, NJ, USA) and 10 ng/mL human recombinant interferon (IFN)-γ (PeproTech). After 6 h, the cells were subjected to quantitative reverse transcription-polymerase chain reaction (qRT-PCR). The culture supernatant was collected 24 h after stimulation and stored at −80 °C for enzyme-link immunosorbent assay (ELISA).

### qRT-PCR

TARC mRNA expression in HaCaT cells was analysed by qRT-PCR. Total RNA was extracted from cells using TRI reagent (Merck Millipore, Darmstadt, Germany) according to the manufacturer’s protocol. The extracted RNA (1 μg) was reverse transcribed in a thermal cycler (PTC-200; MJ Research, Waltham, MA, USA) with 1 mM dNTP, 2.5 U/μL M-MLV reverse transcriptase (Thermo Fisher Scientific, Roskilde, Denmark) and 10 pmol/μL random primer at 42 °C for 50 min. qRT-PCR was performed using 0.5 μg cDNA with SYBR Premix Ex Taq II (Takara Bio, Otsu, Japan) and 10 pmol/μL primers. The primer sequences for human TARC (GenBank: NM_002987.2) were designed as 5′-TTTGAGCTCACAGTGTCACC-3′ (forward) and 5′-GGAGTCTCTGTGTGCAGGTC-3′ (reverse), and were complementary to 18–37 and 110–91, respectively. The primer sequences for human glyceraldehyde-3-phosphate dehydrogenase (GAPDH; GenBank: AF261085.1) were designed as 5′- CTGCTCCTCCTGTTCGACAG-3′ (forward) and 5′- GCGCCCAATACGACCAAATC-3′ (reverse), and were complementary to 33–52 and 151–132, respectively. PCR comprised 1 cycle of preheating (95 °C, 10 min), 40 cycles of denaturation (95 °C, 10 s), and primer annealing and extension (55 °C, 30 s) using an Eco Real Time PCR System (Illumina, San Diego, CA, USA). Results were analysed with the ΔΔCt method using Eco system software (Illumina). The amounts of PCR products were normalised to the expression level of GAPDH mRNA.

### ELISA

IgE level in mice serum and TARC produced by HaCaT cells in culture supernatant were measured using sandwich ELISA. The detailed procedure to measure IgE was described previously [13]. To measure TARC, 50 μL of 2 μg/mL mouse anti-human TARC (R & D Systems, Minneapolis, MN, USA) dissolved in 0.1 M carbonate buffer (pH 10.0) was added to each well of a 96-well Nunc Immuno Plate MaxiSorp (Thermo Fisher Scientific) and incubated at 30 °C for 2 h. Each well was washed three times with phosphate-buffered saline (PBS; pH 7.2) containing 0.05% Tween 20 (PBST) and then post-coated with 300 μL 1% Block Ace (DS Pharma Biomedical, Osaka, Japan) in 0.1 M sodium carbonate buffer (pH 10.0) at 4 °C overnight. After the plates were washed three times with PBST, 50 μL culture supernatant or a standard solution optimally diluted with PBS was added to each well and incubated at 37 °C for 2 h. Recombinant human TARC (Shenandoah Biotechnology, Warwick, PA, USA) was used as the standard. The plates were then washed 3 times with PBST. Next, 100 μL of 4 μg/mL biotinylated goat anti-human TARC (R & D systems) was added to each well and incubated at 37 °C for 30 min. After five washes with PBST, the wells were filled with 100 μL of 2 μg/mL horseradish peroxidase-conjugated streptavidin (BD Biosciences, Franklin Lakes, NJ, USA) and incubated at 37 °C for 30 min. Then, the wells were washed five times with PBST, after which 100 μL TMB Microwell Peroxidase Substrate System (Kirkegaard & Perry Laboratories, Gaithersburg, MD, USA) was added to each well, and the plate was incubated at 30 °C for 15 min. The reaction was stopped by adding 100 μL 1 M phosphoric acid. The absorbance at 450 nm was measured on an iMark microplate reader (Bio-Rad Laboratories, Hercules, CA, USA).

### Statistical analysis

The results obtained in the animal study were analysed using a Mann–Whitney U test. The results obtained in the cell culture study were statistically analysed using a two-tailed Student’s t-test. *P*-values less than 0.05 were considered to indicate a statistically significant difference.

## Results

### Effect of QSEtE treatment on Biostir-AD-induced skin lesions in NC/Nga mice

**Fig. 2:**
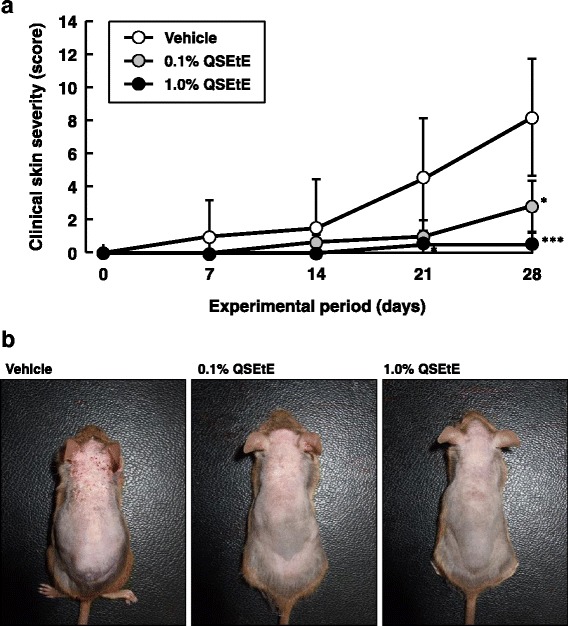
Effect of QSEtE pretreatment on the development of atopic dermatitis-like skin lesions in NC/Nga mice. **a** Clinical skin severity score. Clinical skin severity scores of mice were measured on day 0, and every 7 days after, of the experimental period. Clinical scores are expressed as the mean ± standard deviation (*n* = 6 per group). *P < 0.05 and ***P < 0.001 vs. vehicle group (Mann-Whitney’s U test). **b** Biostir-AD-induced skin lesions in dorsal skin. Photographs were taken on the last day (day 28) of the experimental period

QSEtE was obtained from the seeds of quince with a 0.3% yield on wet weight basis. To examine the effects of QSEtE on AD-like skin lesions in vivo, NC/Nga mice were administered different doses of QSEtE prior to Biostir-AD application. At 1 week after the application of QSEtE, no observable skin irritation was identified. Repeated application of Biostir-AD macroscopically resulted in AD-like lesions on the dorsal skin of mice after 3 weeks. The average clinical severity scores for each group on day 28 were as follows: 8.2 ± 3.9 (vehicle), 2.8 ± 1.7 (0.1% QSEtE), and 0.5 ± 0.8 (1.0% QSEtE) (Fig. 2). Total clinical severity scores in QSEtE-treated groups dose-dependently decreased compared with the scores of vehicle-treated mice. In particular, small lesions of the skin were observed in the 1.0% QSEtE-treated group throughout the entire experimental period. Among the three groups, there were no noticeable adverse effects and no significant differences in body weight gain (data not shown).

**Fig. 3:**
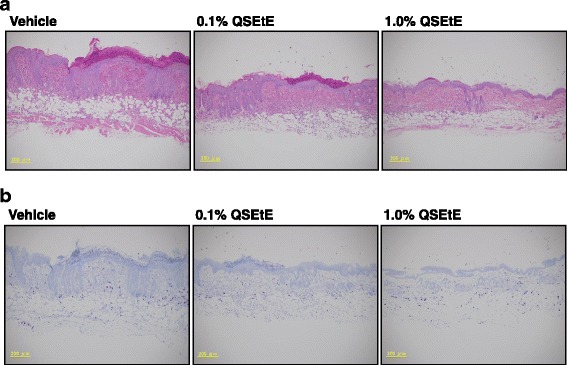
Effect of QSEtE pretreatment on skin lesions in NC/Nga mice. **a** Hematoxylin and eosin-stained dorsal skin lesions. **b** Toluidine blue-stained dorsal skin lesions. The histological images were taken on the last day (day 28) of the experimental period

AD-like symptom, such as epidermal thickening and infiltration of inflammatory cells in the dermis, were observed on day 28 of the experiment (Fig. 3a). QSEtE-treated mice showed a marked reduction in the degree of mast cell infiltration in the skin compared to that reported for the vehicle-treated mice (Fig. 3b).

### Effect of QSEtE on serum total IgE levels of NC/Nga mice

**Fig. 4:**
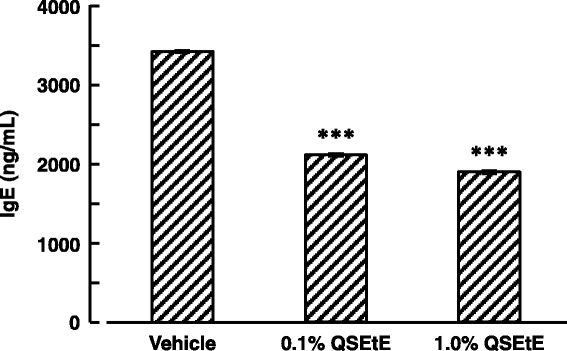
Effect of QSEtE on serum total IgE levels of NC/Nga mice. Blood samples were collected on the last day (day 28) of the experimental period. Data are expressed as the mean ± standard error (*n* = 3). ***P < 0.001 vs. vehicle group (Student’s t-test)

The IgE levels in the serum of mice are shown in Fig. 4. Serum total IgE levels of mice in the vehicle group, 0.1% QSEtE group, and 1.0% QSEtE group were approximately 3418 ng/mL, 2108 ng/mL, and 1895 ng/mL, respectively. IgE levels in 0.1% QSEtE and 1.0% QSEtE group were significantly lower (*P* < 0.01) than those of the vehicle group.

### Effect of QSEtE on TARC expression in the dorsal skin of NC/Nga mice

**Fig. 5:**
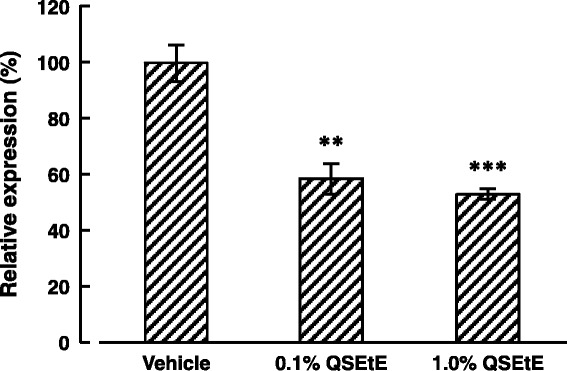
Effect of QSEtE treatment on TARC expression in dorsal skin of NC/Nga mice. Dorsal skin was harvested on the last day (day 28) of the experimental period. Data are expressed as the mean ± standard error (*n* = 3). **P < 0.01 and ***P < 0.001 vs. vehicle group (Student’s t-test)

The TARC expression level in the dorsal skin of mice is shown in Fig. 5. Topical application of QSEtE at the concentrations of 0.1 and 1.0% reduced TARC expression by approximately 41 and 47%, respectively. These reductions were significant in comparison with those observed for the vehicle mice (*P* < 0.01 and *P* < 0.001, respectively).

### Effect of QSEtE treatment on TNF-α- and IFN-γ-induced TARC expression in HaCaT cells

**Fig. 6:**
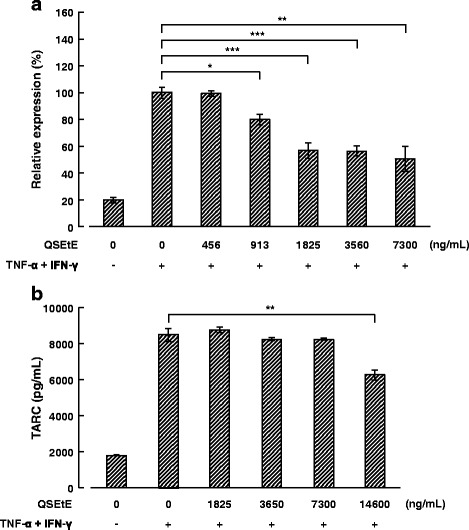
Effect of QSEtE on TNF-α- and IFN-γ-induced TARC expression and production of HaCaT cells. **a** TARC mRNA expression in HaCaT cells treated with TNF-α and IFN-γ (each 10 ng/mL) for 6 h. The expression of mRNA was measured using quantitative reverse transcription-polymerase chain reaction. Values are expressed as the mean ± standard deviation of three independent experiments. *P < 0.05, **P < 0.01, and ***P < 0.001 (Student’s t-test). **b** TARC production in the culture supernatant of HaCaT cells treated with TNF-α and IFN-γ (each 10 ng/mL) for 24 h. Concentrations of TARC were measured using sandwich enzyme-linked immunosorbent assay. Values are expressed as the mean ± standard deviation of three independent experiments. **P < 0.01 (Student’s t-test)

HaCaT cells stimulated with TNF-α and IFN-γ demonstrated increased TARC expression levels compared with those reported for unstimulated cells as described previously [14]. QSEtE (456, 913, 1825, 3650 or 7300 ng/mL) suppressed TNF-α- and IFN-γ-induced TARC expression in a dose-dependent manner (Fig. 6a). Moreover, TARC protein secreted in the supernatant of cells was significantly suppressed by QSEtE at 14,600 ng/mL (*P* < 0.01; Fig. 6b). Under our culture conditions, no significant difference in the proliferation and viability of HaCaT cells was observed at QSEtE concentrations ≤ 40,000 ng/mL (data not shown).

## Discussion

AD often causes a disturbance of the epidermal-barrier function, resulting in dry skin and immune responses against environmental allergens [15]. Various factors are involved in the pathogenesis and development of AD. In particular, house dust mite allergens are considered to be the most common cause of AD [16]. In fact, extracts from *D. farinae*, a common house dust mite, cause AD-like eczematous skin lesions in NC/Nga mice [17]. In the present study, we investigated the effect of QSEtE on house dust mite-induced AD-like skin lesions in NC/Nga mice.

Throughout this study, we demonstrated that QSEtE pre-treatment dramatically reduced subsequently induced dermatitis symptoms (Figs. 2 and 3a), mast cell infiltration (Fig. 3b), and serum IgE level (Fig. 4). In addition, we showed that TARC expression decreased in skin lesions in mice (Fig. 5), and in in vitro cultures of HaCaT cells (Fig. 6).

TARC, also known as CC chemokine ligand 17, is a member of C-C chemokine family and is mainly produced by keratinocytes, dendritic cells, endothelial cells and fibroblasts [18]. Previous studies showed that TARC acts as a chemoattractant that guides Th2 cells that highly express CC chemokine receptor (CCR) 4 and CCR8 [19, 20]. Th2 cells produce chemokines, such as RANTES, a potent chemoattractant for memory-type cluster of differentiation 4 T cells, eosinophils and mast cells, which results in Th2-skewed atopic inflammation [21]. When NC/Nga mice develop AD-like lesions, TARC is overproduced by the keratinocytes in skin lesions, but not in the skin without lesions [22].

In addition, TARC is produced by keratinocytes and has been shown to accelerate inflammatory responses in the skin of human AD patients [23]. In many cases, the serum level of TARC is elevated in patients with AD, and is closely associated with the disease activity of AD [24–26]. Therefore, TARC is recognized as a therapeutic target in the treatment of allergic disorders [27, 28]. Our results suggest the involvement of the suppressive effect of QSEtE on atopic symptoms and TARC production in the skin lesions of NC/Nga mice.

As shown in Fig. 4, we also found that serum levels of IgE were significantly suppressed in the group receiving topical application of QSEtE as compared with that reported for the vehicle-treated group. Although the mechanism of IgE suppression by QSEtE remains unclear, Jung et al. [29] reported that TARC-suppressive phenolic compounds ameliorated AD symptoms accompanied by the suppression of serum IgE. In general, synthesis of IgE by B cells is stimulated by various Th2 cell-derived cytokines such as IL-4, IL-5 and IL-13 [30, 31]. Elevated IgE eventually results in the development of AD-like skin lesions by mediating mast cell degranulation. IgE-associated mast cell degranulation releases inflammatory mediators such as histamine, which increases blood vessel permeability, thus facilitating immune cell migration from the circulation to the affected tissue, resulting in aggravation of AD symptoms [32]. Therefore, we hypothesize that the suppression of TARC in lesion site by QSEtE may suppress the of mast cell recruitment and improve Th1/Th2 immune balance to downregulate IgE production, resulting in the alleviation of IgE- and mast cell-related atopic symptoms. In this study, we did not determine the components responsible for the anti-atopic effect of QSEtE. However, it should be noted that QSEtE could easily pass through the sterilizing filter easily, which was different from the property of water-soluble quince seed mucilage. Additional studies are needed to identify the primary active components of QSEtE

## Conclusions

In conclusion, we showed that the topical application of QSEtE reduced the development of house dust mite allergen-induced skin lesions in NC/Nga mice. In addition, in vitro evaluations showed that QSEtE directly suppressed TARC expression and the production of keratinocytes. Although the results obtained from NC/Nga mice would be difficult to directly extrapolate to humans owing to genetic heterogeneity of humans, the ability of QSEtE to suppress TARC from keratinocytes may have a role in its effectiveness for treating AD. We believe that QSEtE could be a good candidate to protect the skin from allergen-induced Th2-type inflammation in the onset and development of AD.

## Abbreviations

AD: Atopic dermatitis
CCR: CC chemokine receptor
GAPDH: Glyceraldehyde-3-phosphate dehydrogenase
QSEtE: Ethanol extract of quince seeds
TARC: Thymus and activation-regulated chemokine
